# Bio-control agents activate plant immune response and prime susceptible tomato against root-knot nematodes

**DOI:** 10.1371/journal.pone.0213230

**Published:** 2019-12-03

**Authors:** Sergio Molinari, Paola Leonetti

**Affiliations:** Institute for Sustainable Plant Protection, National Research Council of Italy (IPSP-CNR), Bari, Italy; USDA-ARS, UNITED STATES

## Abstract

Beneficial microorganisms are generally known to activate plant defense against biotic challenges. However, the molecular mechanisms by which activated plants react more rapidly and actively to pests remain still largely unclear. Tomato plants pre-treated with a mixture of beneficial bio-control agents (BCAs), as soil-drenches, were less sensitive to infection of the root-knot nematode (RKN) *Meloidogyne incognita*. To unravel the molecular mechanisms of this induced resistance against RKNs, we used qRT-PCR to monitor the expression, in tomato roots and leaves, of 6 key defense genes. Gene transcripts were detected until the 12^th^ day after BCA treatment(3, 7, 8, 12 dpt) and3 and 7 days after nematode inoculation of pre-treated plants. Early after BCA treatment, the salicylic acid (SA)-dependent pathogenesis related gene (*PR*-gene), *PR-1b*, marker of the systemic acquired resistance (SAR), was systemically over-expressed. Another *PR*-gene, *PR-5*, was over-expressed at later stages of BCA-plant interaction, and only in roots. Activation of defense against RKNs was attested by the early up-regulation of 4 genes (*PR-1*, *PR-3*, *PR-5*, *ACO*) in pre-treated plants after inoculation. Conversely, the expression of the JA/ET-dependent gene *JERF3* did not increase after nematode inoculation in primed plants. A catalase gene (*CAT*)was highly over-expressed by nematode infection, however, this over-expression was annulled at the earliest stages or limited at the later stages of infection toBCA-treated roots. Enzyme activities, such as glucanase and endochitinase, were enhanced in roots of pre-treated inoculated plants with respect to plants left not inoculated as a control. These findings indicate that BCA interaction with roots primes plants against RKNs. BCA-mediated immunity seems to rely on SA-mediated SAR and to be associated with both the activation of chitinase and glucanase enzyme activities and the inhibition of the plant antioxidant enzyme system. Immunity is triggered at the penetration and movements inside the roots of the invading nematode juveniles but probably acts at the feeding site building stage of nematode infection.

## Introduction

Bio-control agents (BCAs) are beneficial soil-borne micro-organisms that interact with roots and improve plant health. These root-associated mutualists can be divided into three main groups: Bio-Control Fungi (BCF), Arbuscular Mycorrhizal Fungi (AMF), and Plant Growth Promoting Rhizobacteria (PGPR) [1, 2]. BCF include the well-studied *Trichoderma* spp., a class of opportunistic fungi that may colonize roots of most plants, reducing the infection of plant pathogens and parasites and promoting positive responses in stressed plants [3]. AMF are obligate root symbionts, diffused in most of the soils, that improve plant growth and can alleviate both abiotic and biotic plant stresses [4]. Several genera of the rhizosphere bacteria, such as *Pseudomonas* spp., *Bacillus* spp., and *Streptomyces* spp., can enhance plant growth and improve health [5]. BCAs can control pests and diseases by activation of plant immune system [6].

Immune response in plants is regulated by several low molecular weight molecules known as phytohormones, i.e. salicylic acid (SA), jasmonic acid (JA) and ethylene (ET). Furthermore, phytohormones regulate many aspects of plant life, as well, such as reproduction and seed production, photosynthesis, flowering, and response to environmental abiotic challenges. BCAs adopt several sophisticated molecular mechanisms to activate plant immune response against pathogen and parasite attacks. One of the most studied mechanism is recognized as systemic acquired resistance (SAR), which is otherwise triggered by local infections causing tissue necrosis [7]. SAR provides long-term resistance to (hemi)biotrophic pathogens and pests, is correlated with the activation of Pathogenesis Related (*PR*-) genes, and is mediated by SA. Rhizobacteria-induced systemic resistance (ISR) is regulated by JA and ET, is not associated with changes in *PR*-gene expression, and is mainly effective against necrotrophic pathogens and herbivorous insects [1, 5].

BCF-induced plant resistance has extensively been described, although the signaling elicited seems to vary according to the considered beneficial fungus and the elicited plant species [2]. In a recent study on the interaction of two *T*. *harzianum* strains (T908, T908-5) with tomato plants, the expression of SAR-marker genes was markedly repressed as soon as 24 h after fungal inoculation; however, subsequent inoculation with root-knot nematodes (RKNs) caused an over-expression of the same genes [8]. Tissue preconditioning to realize a more effective defense after a/biotic challenges is a suitable strategy that plants generally adopte to save the costs of a permanent activated state, a phenomenon known in literature as priming [9]. Accordingly, some *Trichoderma* spp. probably prime plants for SAR, but the entire pathway is maintained unexpressed until a subsequent pathogen/parasite attack occurs. The same events were reported to occur in cucumber primed by *T*. *asperellum* (T203) against *Pseudomonas syringae* pv. *lachrymans* [10]. Priming for defense seems to be induced also by AMF [11].

AMF produce the so-called mycorrhiza-induced resistance (MIR), acting against numerous different pathogens [11]. MIR has mostly been studied against necrotrophs, generalist chewing insects, and pathogenic fungi [11, 12]. When AMF were used against those pests, JA-signaling pathway was reported to be essential to MIR [12]. However, MIR, like SAR, acts also through SA-dependent defenses giving protection against (hemi)biotrophic pathogens and parasites, like plant parasitic nematodes (PPNs). The elicitation of JA-signaling pathway found in MIR activation may depend on an ISR component elicited by the rhizobacteria normally present in the mycorrhizosphere [4]. On the other hand, recent investigation proves that SAR and ISR often overlap, with crosstalk taking place between the relative pathways [1]. Few studies have investigated the molecular mechanisms underlying the systemic resistance induced by AMF against PPNs. Generally, a putative major role of JA-dependent pathway in MIR against PPNs has been questioned and needs confirmation [13].

RKNs are obligate soil-borne animal parasites of almost all crops world-wide. They cause significant damages to the attacked crops, and the consequent decrease in both yield and quality leads to economic losses estimated in more than €80 billion/year in worldwide agriculture [14]. RKNs enter the roots as motile second-stage juveniles (J2s), and move intercellularly through the elongation zone to reach some few cortical cells which are thus transformed into discrete giant or nurse cells. Throughout their life cycle, nematodes maintain these elaborate feeding sites that principally serve to actively transfer solutes and nutrients to the developing nematode. J2ssoon become sedentary and, through two molts as J3 and J4, develop into adult gravid females. Females parthenogenetically reproduce by laying 200–400 eggs in an external gelatinous matrix, that is clearly visible outside the roots as an egg mass. Moreover, nematode action induces hypertrophy and hyperplasia of the surrounding tissues, thus causing the formation of the familiar galls on roots [15]. RKNs produce several proteins in the esophageal glands that are introduced, via the stylet, into root cells, or transferred to the root apoplasm by secretion from cuticlin or amphids. An increasing amount of reports has shown that most of these proteins are effectors that contribute to plant defense suppression during infection [16]. Control of plant parasitic nematodes is generally difficult and, at present, still relies on the use of chemical toxic nematicides on cash crops. Such large use is increasingly being banned by European Union Directives, with the aim to reduce pesticide contamination of soils and food. Therefore, scientists are looking for alternative low-impact methods of nematode control, such as genetic and induced resistance, or the use of biocontrol agents [17, 18, 19].

Many reports have shown that beneficial root endophytes, such as *Trichoderma* and G*lomus* spp., can reduce infections of endoparasitic nematodes through elicitation of the plant immune system [8,20, 21]. Rhizobacteria belonging to specific strains of *Pseudomonas* spp. have long been known to be effective in reducing RKN infection through elicitation of ISR [22]. More recently, three strains of *Bacillus subtilis* and one of *Rhizobium etli*, antagonists also of fungal pathogens, have been reported to reducethe number of both galls and egg masses in roots of tomato plants inoculated with RKNs by eliciting ISR [23].

A mixture of AMF, BCF and PGPR was used in this study as a pre-treatment of tomato plants before inoculation with *M*. *incognita*. Transcriptional analysis of genes and activity tests of key enzymes involved in plant defense were used to have information on the molecular pathways involved in the activation of plant immune system against these soil-borne parasites. We monitored the expression of six genes from both leaves and roots and the activity of three enzymes from roots, all involved in defense response. Detection of gene expressions were performed at 3, 7, 8, and 12 days after treatment (dpt) and 3–7 days after inoculation (dpi) with nematodes. Therefore, we detected the early response of plants to colonization of beneficial microorganisms, and the priming process that such colonization induces against the subsequent RKN attack. Data of this paper confirm that plant defense against RKNs was activated by the used BCAs, and was basically characterized by the over-expression of *PR*-genes.

## Materials and methods

### Treatments of tomato plantswith BCAs

Seeds of the tomato (*Solanum lycopersicum* L.) cultivar Roma VF, susceptible to root-knot nematodes (RKNs), were surface-sterilized and sown in river sand (previously sterilized by autoclaving twice at 121 °C for 30 min). Seedlings were transplanted to 110-cm^3^ clay pots, filled with 150 g of sterilized river sand. Pots were put in temperature-controlled benches (soil temperature 23–25°C), located inside a glasshouse. Plantlets were provided with a regular regime of 12 h light/day, periodically watered and weekly fertilized with Hoagland’s solution. Plants were allowed to grow to the 4–6 compound leaf stage. Before treatments, average fresh weights of plants were measured; young plants with a weight ranging3-4 g were selected. BCAs contained in Micosat F^®^ (named Myco in the text), a commercial product by C.C.S. Aosta, Italy, were provided to plants at the dosage of 0.2 g product per g plant fresh weight (0.6–0.8 g/plant). One gram Mycois constituted by 40% roots hosting arbuscular mycorrhiza forming fungi of *Glomus* spp. (*Glomus* spp. *GB 67*, *G*. *mosseae GP11*, *G*. *viscosum GC 41*) and 12.4 x 10^7^ C.F.U. of a mixture of antagonistic fungi (*Trichoderma harzianum TH 01*, *Pochonia chlamydosporia PC 50*), rhizo-bacteria such as *Agrobacterium radiobacter AR 39*, *Bacillus subtilis BA 41*, *Streptomyces* spp., and yeasts (*Pichia pastoris PP* 59). Myco powder was dissolved in a peptone-glucose suspension (0.7 g ml^-1^), and incubated in an orbital shaker at 25°C for 3 days in dark. In some experiments, 100 μg ml^-1^ Amphotericin B, a potent antifungal compound, was added to the suspension to exclude the effect on plants of the fungal components of the mixture. Then, groups of plants were soil-drenched with suitable amounts of Myco suspension, whilst control plants were provided with the sole peptone-glucose suspension.

### Inoculation of tomato plants with nematodes

Populations of the root-knot nematode *Meloidogyne incognita* (Kofoid*et* White) Chitwood, collected from field and reared in a glasshouse on susceptible tomato, were used for plant inoculation. Females of such a population were identified as *M*. *incognita* by electrophoretic esterase and malate dehydrogenase isozyme patterns [24]. Invasive second-stage juveniles (J2s) were obtained by incubation of egg masses in tap water at 27°C; 3-day-old J2s were collected and used for inoculation. Five days after Myco treatment, groups of 6 treated and untreated plants were inoculated with 300 J2/plant, whilst other groups were left not inoculated. Inoculation was carried out by pouring 2–4 ml of J2 stirring suspensions into 2 holes made in the soil around the plants. Detection of nematode infection was performed 3, 7, 21, and 40 dpi. Plants were grown in pots filled with sterilized river sand in the experiments in which harvest was predicted to occur 3 and 7 days after nematode inoculation; conversely, plants were grown in pots filled with a mixture of sterilized loamy soil and sand (1:1,v:v) when harvest was predicted at 21 and 40 dpi.

### Detection of nematode infection

The numbers of motile vermiform individuals (second stage, J2s) and sedentary swollen individuals (third and fourth stages, sedentary juveniles, SJs) that had, respectively, penetrated and established into the roots 3 and 7 dpi were determined under a stereoscope after coloration by the sodium hypochloride-acid fucsin method [25]. In the roots harvested 21 and 40 dpi, only adult reproducing females and egg masses were searched and counted. Extraction of swollen females from roots was carried out by incubation with pectinase and cellulase enzyme mixture at 37° C in an orbital shaker to soften the roots. After a brief homogenization in physiological solution, females were collected on a 90 μm sieve and counted under a stereoscope (x 12 magnification). Egg masses (EMs) were colored by immersing, the roots in a solution (0.1 g L^-1^) of the colorant Eosin Yellow, at least for 1 h in a refrigerator; red-colored EMs were then counted under a stereoscope (x 6 magnification). Samples were arranged from roots of 2 plants; root samples were weighed before extractions or colorations. The numbers of nematode stages were expressed per g root fresh weight. Additionally, shoot and root weights of treated and untreated inoculated plants were measured after harvest.

### RNA extraction and quantitative real-time reverse PCR

Tissues (leaves and roots) from untreated and Myco-treated plants were collected 3, 7, 8, and 12 dpt. Tissues from untreated and Myco-treated plants, inoculated with nematodes, were collected 3 and 7 dpi. Tissue samples were weighed and stored at -80°C, if not immediately used for RNA extraction. Plants came from 2 independent bioassays; per each bioassay, 3 different tissue samples were collected per treatment. RNA extraction was carried out on each collected samples. Plant tissues were separately ground to a fine powder in a porcelain mortar in liquid nitrogen. An aliquot of macerated tissue (100 mg per sample) was used for RNA extraction. Extractions of total RNA were carried out using an RNA-easy Plant Mini Kit (Qiagen, Germany), according to the instructions specified by the manufacturer. RNA quality was verified by electrophoresis runs on 1.0% agarose gel and quantified using a Nano-drop spectrophotometer. QuantiTect Reverse Transcripton Kit (Qiagen, Germany) with random hexamers was used for cDNA synthesis, from 1 μg of total RNA. PCR mixtures (20-μl final volume)contained RNAse free water, 0.2μM each of forward and reverse primers, 1.5μl cDNA template and 10 μlSYBR^®^ Select Master Mix (Applied Biosystems, Italy). PCR cycling consisted in an initial denaturation step at 95 °C (10 min); 40 cycles at 95 °C (30 s), at 58 °C (30 s), at 72 °C (30 s), with the final step at 60 °C (1 min).

qRT-PCRs were performed in triplicate, using an Applied Biosystems^®^ StepOne^™^ instrument. Actin was used as the reference gene, since its expression in tomato tissues has been proved not to vary after infestation by nematodes. The GenBank accession used for PR-1 was described as *PR-1b* (*P6*) in [26]. Primers for the analyzed genes are described in Table 1. The relative fold changes in gene expression was calculated by the 2^-ΔΔCT^ method [27].

**Table 1:** Tomato defense-related genes examined in this study and the specific primers used in quantitative reverse transcriptase-polymerase chain reaction (qRT-PCR).

Gene	Accession number	Protein Activity	Primer sequence (5'-3')
*PR1-1b*	NM_001247385.2	unknown	F:GATCGGACAACGTCCTTAC R:GCAACATCAAAAGGGAAATAAT
*PR-3*	NM_001247474.2	chitinase	F:AACTATGGGCCATGTGGAAGA R:GGCTTTGGGGATTGAGGAG
*PR-5*	NM_001247422.3	thaumatin-like	F:GCAACAACTGTCCATACACC R:AGACTCCACCACAATCACC
*JERF3*	NM_001247533.2	Jasmonate Ethylene Response Factor 3	F:GCCATTTGCCTTCTCTGCTTC R:GCAGCAGCATCCTTGTCTGA
*ACO*	XM_015225653.2	1-aminocyclopropane-1-carboxylic acidoxidase	F:CCATCATTTCTCCAGCATCA R:TTGGCAGACTCAAATCTAGG
*CAT*	NM_001247257.2	catalase 2	F:TGCTCCAAAGTGTGCTCATC R:TTGCATCCTCCTCTGAAACC
*actin*	NM_001321306.1	actin-7-like	F:GATACCTGCAGCTTCCATACC R:GCTTTGCCGCATGCCATTCT

### Protein extraction and enzyme activity assays

Proteins were extracted from roots of plants at8 and 12 dpt and at 3 and 7 dpi. Roots were set free from sand, and thoroughly rinsed with tap water. Roots and leaves were separated from shoots. Roots from untreated and Myco-treated plants were collected, dried, weighed and put on ice. Root samples were immediately used for protein extractions or stored at -80°C. Samples were ground in porcelain mortars by immersion in liquid nitrogen. For each bioassay, three different powdered samples of roots, coming from 6 plants per treatment, were produced and suspended in a grinding buffer (1:5, w:v) of 0.1 M potassium phosphate buffer (pH 6.0),added with 4% polyvinylpyrrolidone and the protease inhibitor phenyl-methane-sulfonyl fluoride (PMSF, 1 mM). Suspensions were further ground using a Polytron^®^ PT–10–35 (Kinematica GmbH, Switzerland), and filtered through four layers of gauze. Filtrates were centrifuged at 12000 x *g* for 15 min. Supernatants were filtered through 0.45 μm nitrocellulose filters applied to 10-ml syringes. These filtrates were ultra-filtered at 4°C through 20-ml Vivaspin micro-concentrators (10,000 molecular weight cut off, Sartorius Stedim, Biotech GmbH, Germany). Retained protein suspensions were used for protein content and enzyme assays. Protein content was determined by the enhanced alkaline copper protein assay, with bovine serum albumin as the standard [28].

Chitinase activity (CHI) was measured by a colorimetric procedure that detects N-acetyl-D-glucosamine (NAG) [29]. The hydrolytic action of chitinase produces chitobiose which is converted into NAG by the β-glucuronidase introduced in the reaction mixture. Suspended chitin (250 μl, 10 mg/ml) from shrimp shells (Sigma-Aldrich, Italy) was added to 50 μl of leaf extract or 100 μl of root extract diluted in 200–150 μl of 0.05 M sodium acetate buffer (pH 5.2) containing 0.5 M NaCl. The reaction was allowed by incubating the mixtures in eppendorfsfor 1 h at 37°C in an orbital incubator, and stopped by boiling at 100°C for 5 min in a water bath. Eppendorfs were centrifuged at 10000 x *g* for 5 min at room temperature. Supernatants (300 μl) were collected and added with 5 μl β-glucuronidase (Sigma, type HP-2S, 9.8 units/ml). Reaction on/off was carried out as previously described; reaction mixtures were let cool at room temperature. After adding 60 μl of 0.8 M potassium tetraborate (pH 9.1), mixtures were heated to 100°C for 3 min and cooled to room temperature. Then, 1% 4-dimethylaminobenzaldehyde (1.2 ml, DMAB, Sigma) was added, and mixtures incubated at 37°C for 20 min. Absorbance was read at 585 nm (DU-70, Bechman), and the amount of produced NAG was determined by means of a standard curve obtained with known concentrations (4.5–90 nmoles) of commercial NAG (Sigma). Blanks (negative controls) were mixtures in which tissue extracts were not added; positive controls were arranged by adding 10 μl chitinase from *Streptomyces griseus*(Sigma, 200 units/g). The assay was conducted on 6 samples per treatment, and chitinase expressed as nanokatal per mg protein (nkat/mg prot), with 1nkat defined as 1.0 nmol NAG produced per second at 37°C.

β-1,3-Endoglucanase (glucanase, GLU) activity was measured by determining the amount of glucose released from laminarin (Sigma, Italy) used as substrate. Reaction mixtures consisted in laminarin (0.4 mg) and 100 μl tissue extracts in 300 μl 0.1 M sodium acetate (pH 5.2) that was incubated at 37°C for 30 min. After incubation for glucose production, Nelson alkaline copper reagent (300 μl) was added and the mixtures kept at 100°C for 10 min. Once mixtures had cooled at room temperature, Nelson chromogenic reagent (100 μl) was added for reducing sugars assays [30]. Negative and positive controls consisted of grinding buffer and laminarinase (2 U/ml), respectively. Enzyme activity was expressed as μmol glucose equivalents released per minute, according to a standard curve created with known amounts (10–200 μg ml^-1^) of commercial glucose (Sigma, Italy).

Ascorbate peroxidase activity (APX) was determined as the rate of disappearance of ascorbate in presence of hydrogen peroxide [31]. Reaction mixtures contained 0.1 M TES, pH 7.0, 0.1 mM EDTA, 1 mM ascorbate, 0.1 mM H_2_O_2_, 10–20 μl root extracts, in 0.5 ml final volume. Decrease in absorbance at 298 nm was monitored in a double-beam spectrophotometer (PerkinElmer 557) and indicated ascorbate oxidation; 1unit of enzyme expressed the oxidation of 1 μmole ascorbate per min (ε = 0.8 mM^-1^ cm^-1^).

### Statistical analysis

Means of values ± standard deviations of nematode stages found into the roots were calculated by 9 replicates (n = 9), coming from 3 different experiments, arranged in 6 plants per treatment. Weight values of roots and shoots are means ± standard deviations from 18 replicates (n = 18). Means from untreated and Myco-treated plants were separated by a paired *t*-test (*P<0.05; **P<0.01). As it concerns qRT-PCR data, means ± standard deviations of 2^-ΔΔCt^ values of each group from untreated and Myco-treated tissues (n = 6) were separated by the non-parametric Kolmogorov-Smirnov test (*P<0.05) (S1 Table). Furthermore, enzyme activity values, means ± standard deviations were the result of 9 replicates (n = 9). Nine tissue samples were obtained from 3 different bioassays. Moreover, each value was calculated on the basis of 3 repeated spectroscopic measurements on each protein extract. Values of enzyme activities were expressed as units mg^-1^ protein; means were separated by a paired *t*-test (*P<0.05; **P<0.01).

## Results

### BCAs activate the immune response of tomato plants

Expression of six genes involved in defense to biotic challenges were detected by qRT-PCR in roots and leaves of plants 3, 7, 8, and 12 days after treatment with the BCAs contained in Myco, a commercial mixture of AMF, BCF, and PGPR. At first,3genes belonging to the *PR-1*, *PR-3*, and *PR-5* gene families, were tested. The *PR1-P6* or *PR-1b1* gene tested (Acc. n. NM_001247385.2) encodes for low molecular-weight proteins of unknown biochemical function. We chose to test *PR-1b1* gene expression because it was found to be strongly activated during the hypersensitive response (HR) to pathogens in tomato, whilst the other gene of the family, *PR-1a2*,was not induced by pathogenic signals [32]. The *PR-3* gene family encodes for several types of endochitinases, and has generally been reported to be induced by activation of JA-signaling pathway and ethylene treatments in tomato [33]. The *PR-5* gene family encodes for thaumatin-like proteins and is involved in osmotic regulation of cells. Expression of *PR-1* is highly induced by SA treatment to plants and over-expressed in SAR against biotrophic pathogens [34, 35].

**Fig 1:**
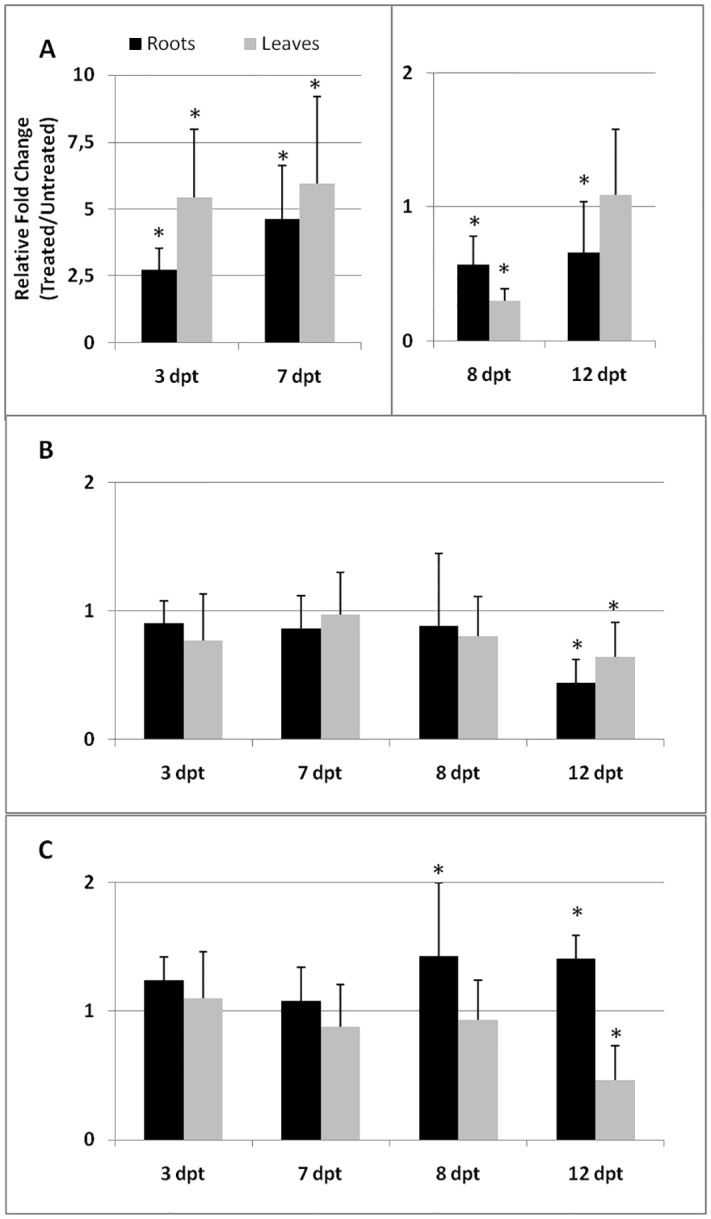
Expression of *PR-1b*, *PR-3*, and *PR-5* genes in tomato tissues after treatment with BCAs. Expression of *PR-1b* (A), *PR-3* (B) and *PR-5* (C) genes was detected by quantitative real-time reverse-transcription polymerase chain reaction (qRT-PCR) in roots and leaves of susceptible tomato plants at 3, 7, 8, and 12 days post treatment (dpt) with BCAs. Data are the mean fold changes (*n* = 6)± SD in gene transcript levels of tissues from BCA-treated plants relative to those from untreated control plants (the value 1 indicates no change). An asterisk (*) indicates that the mean fold change is significantly different from 1 as determined by the non-parametric Kolmogorov-Smirnov test (*P*<0.05).

Expression of *PR-1b* gene was systemically highly activated in BCA-treated plants, as soon as 3 and 7 dpt. After this early activation, *PR-1b* gene expression in treated plants was found to be repressed with respect to untreated plants(Fig 1A). No significant changes in *PR-3* gene (Acc. n. NM_001247474.2) expression between untreated and treated plants were observed up to 8 dpt; at 12 dpt, a significant inhibition of the gene expression was detected in both roots and leaves due to BCA treatment (Fig 1B). Activation of *PR-5* gene (Acc. n. NM_001247422.3) expression was delayed to 8–12 days after BCA treatment and occurred only in roots (Fig 1C); conversely, in leaves, *PR-5* gene was significantly down-loaded in the latest stage of the experimental period.

**Fig 2:**
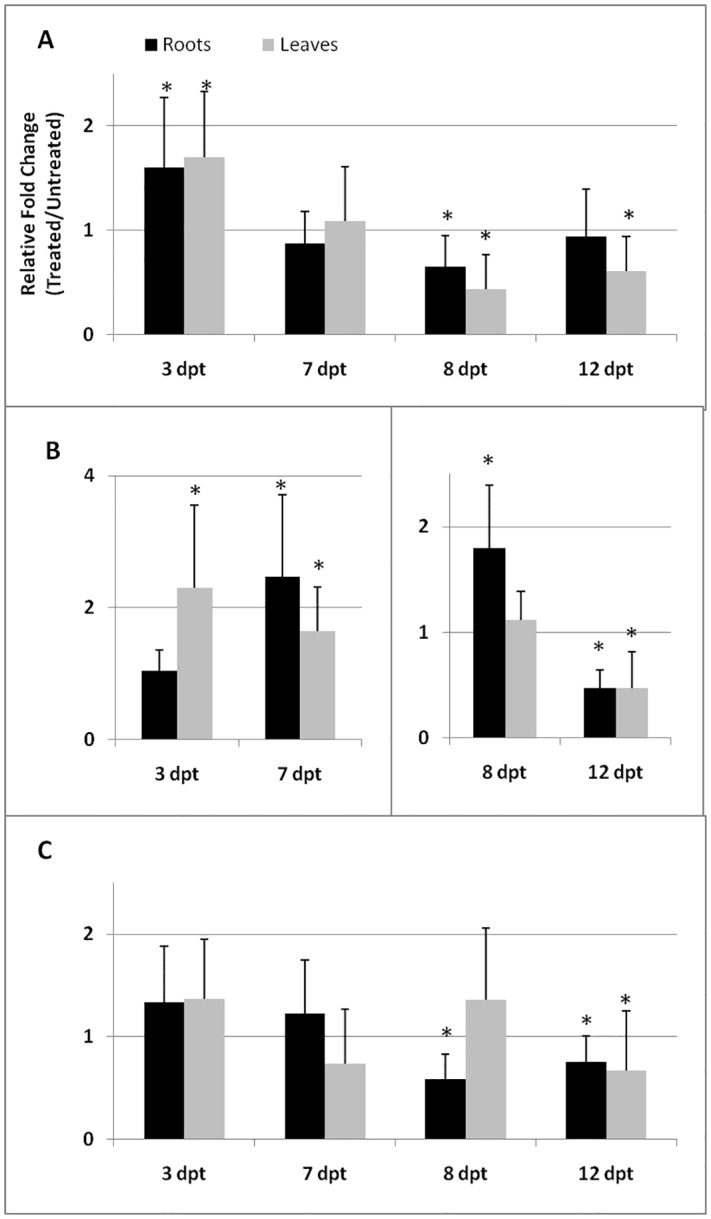
Expression of *JERF3*, *CAT*, and *ACO* genes in tomato tissues after treatment with BCAs. Expression of *JERF3* (A), *CAT* (B) and *ACO* (C) genes was detected by quantitative real-time reverse-transcription polymerase chain reaction (qRT-PCR) in roots and leaves of susceptible tomato plants at 3, 7, 8, and 12 days post treatment (dpt) with BCAs. Data are the mean fold changes (*n* = 6)± SD in gene transcript levels of tissues from BCA-treated plants relative to those from untreated control plants (the value 1 indicates no change). An asterisk (*) indicates that the mean fold change is significantly different from 1 as determined by the non-parametric Kolmogorov-Smirnov test (*P*<0.05).

The second series of 3tested genes included *JERF3*, *CAT*, and *ACO*. *JERF3* gene (Acc. n. NM_001247533.2) encodes for a member of ERF proteins, a trans-acting factor responding to both ET and JA in tomato [36]. *ACO* gene (Acc. n. XM_015225653.2) encodes for ACC oxidase, the enzyme which catalyzes the last step of ET biosynthesis, whilst *CAT* gene (Acc. n. NM_001247257.2) encodes for a catalase, which neutralizes the toxic hydrogen peroxides produced in plant defense against pathogens and parasites. *JERF3* gene was significantly downloaded in roots at 8 dpt and in leaves at 8 and 12 dpt from BCA-treated plants, after a slight systemic induction at 3 dpt (Fig 2A). *CAT* gene was generally over-expressed until 8 dpt by BCA treatment, that, conversely, repressed its expression at 12 dpt (Fig 2B) Expression of *ACO* gene was not affected in the earliest days after treatment with BCAs; however, this gene appears to be down-loaded by BCA treatment at 8 and 12 dpt (Fig 2C).

### BCAs prime tomato plants against root-knot nematodes

The amount of motile invasive J2sinto the roots at 3 and 7 dpi was not significantly affected by BCA treatment. However, feeding site construction is the early step of infection, at which motile J2s become sedentary, start to grow, and transform cortical cells into nursery cells; feeding sites transfer nutrients from plant metabolism to the developing nematodes. At 7 dpi, sedentary juveniles extracted from roots of BCA-treated plants were one third of those from untreated plants. At 21 dpi, BCA treatment highly reduced the reproducing females and egg masses present in/on roots. At the end of life cycle of successfully developed nematodes (40 dpi), females and egg masses in roots of BCA-treated plants were still significantly lower than in roots of untreated plants, although at a minor extent. When Myco suspensions were added with the potent antifungal compound Amphotericin B, the suppressive effect of the BCA mixture on nematode infection was inverted; inactivation of the fungal components resulted in a significant augment of females and egg masses in BCA-treated with respect to untreated plants (Table 2).

**Table 2:** Nematode individuals penetrated, developed and reproduced in roots of tomato plants untreated and treated with BCAs at different days after inoculation (dpi).

average no. per plant ± stdev							average no. per g root fresh weight					
dpi	Shoot Weight (g)		Root Weight (g)		Motile invasive J2s		Sedentary J3-4 forms		Females		Egg masses	
	Untreated	Treated	Untreated	Treated	Untreated	Treated	Untreated	Treated	Untreated	Treated	Untreated	Treated
3	2.5±0.6	2.4±0.5	0.4±0.2	0.4±0.2	18±10	14±10	0	0	0	0	0	0
7	3.3±0.8	3.3±0.6	0.4±0.2	0.4±0.2	146±74	112±84	24±12	8±7*	0	0	0	0
21	4.8±1.7	4.9±1.3	1.2±0.6	1.1±0.8	nd^b^	nd	nd	nd	28±12	6±4*	12±5	2±2*
40	9.4±3.6	8.9±3.8	1.8±1.0	2.2±1.3*	nd	nd	nd	nd	155±28	83±10*	97±28	52±14*
40+AMPHO^a^	11.4±2.5	11.7±2.5	1.8±0.8	1.9±0.8	nd	nd	nd	nd	169±67	378±155*	102±33	168±18*

* significantly different (P<0.05) according to a paired *t*-test;

^a^tests in which BCA suspension was added with 100 μg ml^-1^ Amphotericin B; nd = not determined.

**Fig 3:**
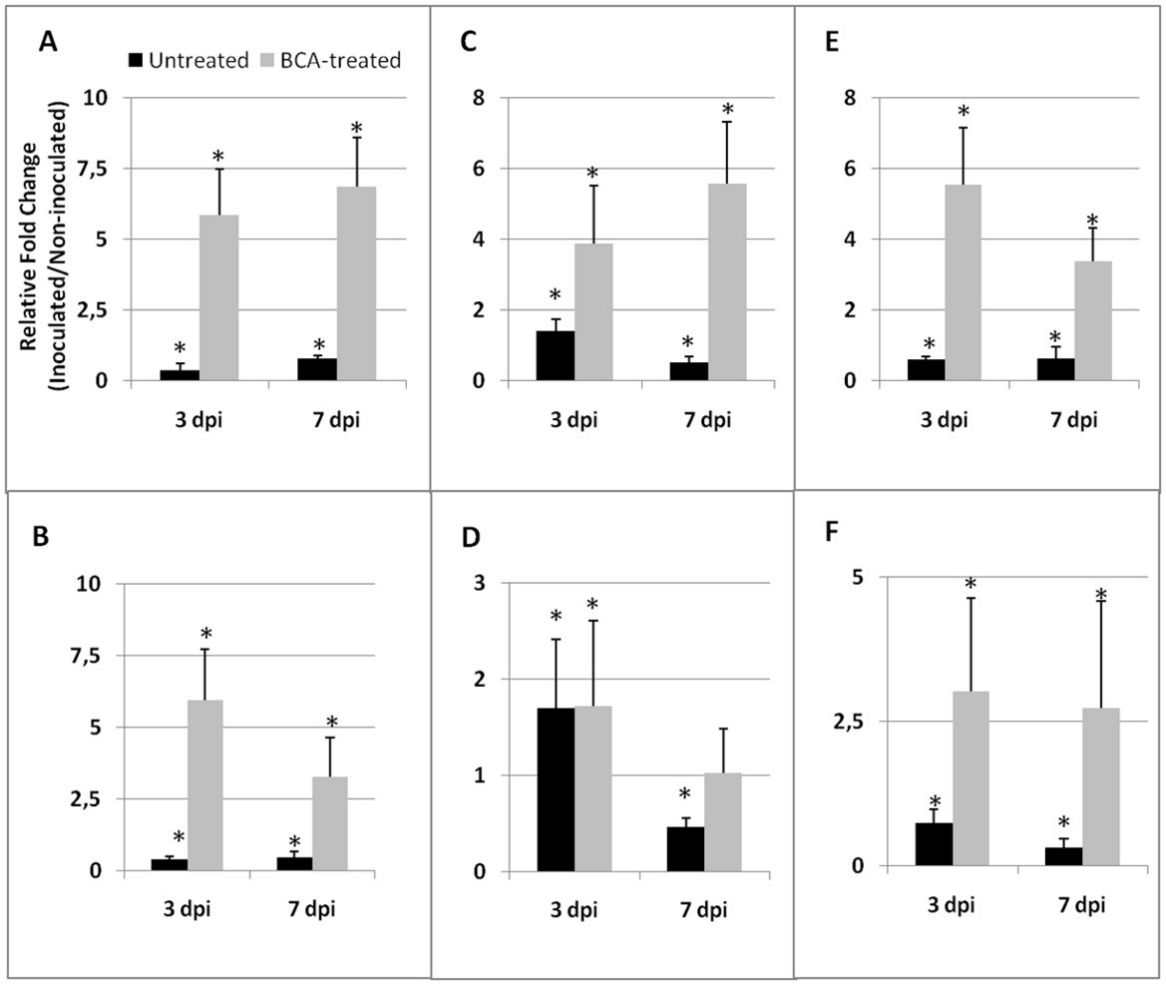
Expression of *PR-1b*, *PR-3*, and *PR-5* genesin tomato tissues of BCA-pretreated plants after inoculation with RKNs. Expression of *PR-1b*, *PR-3*, and *PR-5* genes was detected by quantitative real-time reverse-transcription polymerase chain reaction (qRT-PCR) in roots (A, C, and E, respectively)and leaves (B, D, and F, respectively)of nematode-infested susceptible tomato plants, untreated and BCA-pretreated,3 and 7 days after inoculation (dpi) with 300 J2*M*. *incognita*. Data are the mean fold changes (n = 6) ±SD in gene transcript levels of tissues from inoculated plants compared with tissues from non-inoculated control plants (the value of 1 indicates no change). An asterisk (*) indicates that the mean fold change is significantly different from 1 as determined by the non-parametric Kolmogorov-Smirnov test (P<0.05).

BCA-induced priming is indicated by the over-expression of *PR*-genes at 3 and 7 days after nematode inoculationof pre-treated plants. Gene over-expression involved all the tested *PR*-genes (*PR-1b*, *PR-3*, and *PR-5*), and was systemic, except for *PR-3* in leaves at 7 dpi(Fig 3). Nematode infection in plants not treated with BCAs generally and systemically repressed *PR*-mediated plant immunity; only *PR-3* gene expression at 3 dpi was systemically induced (Fig 3C and 3D).

**Fig 4:**
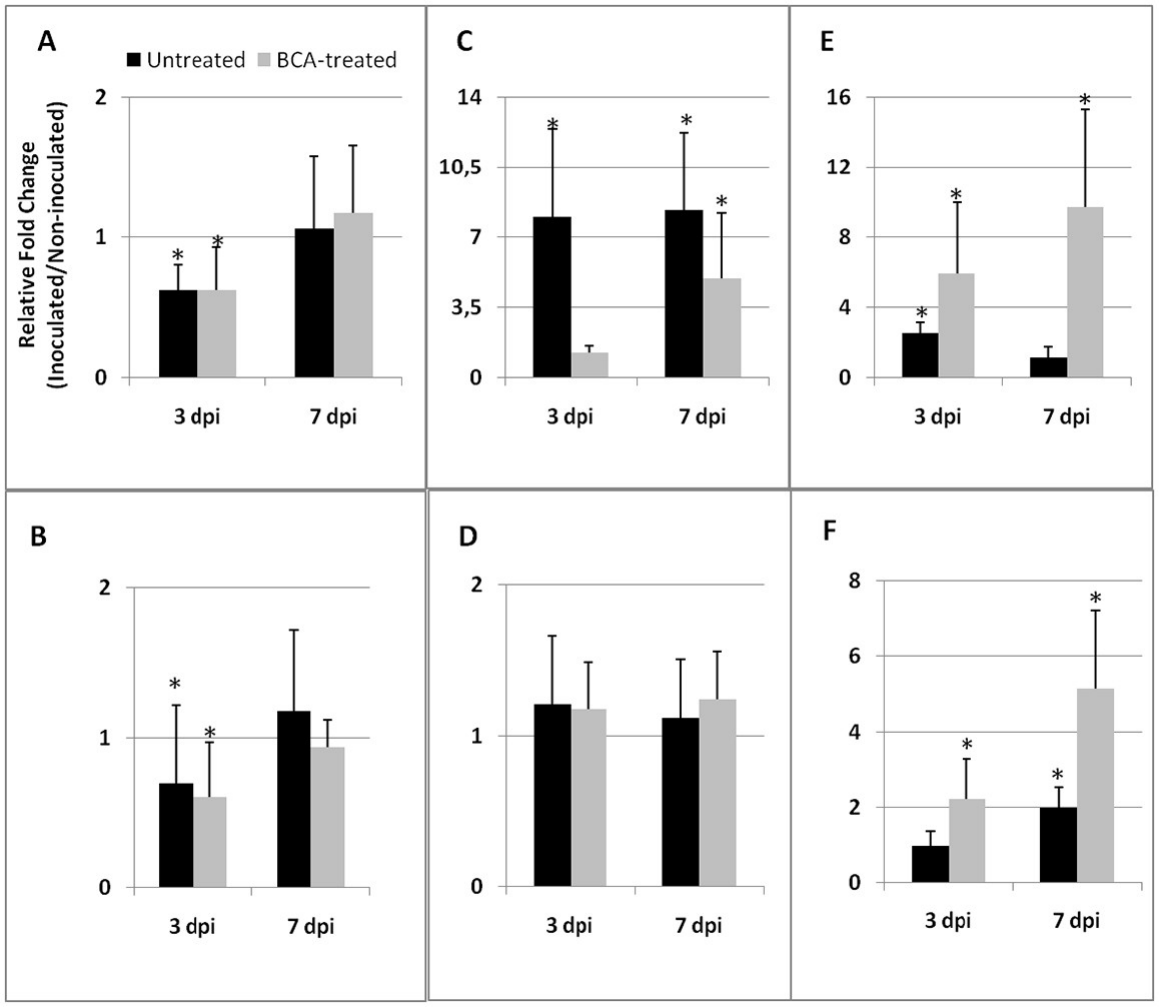
Expression of *JERF3*, *CAT*, and *ACO* genesin tomato tissues of BCA-pretreated plants after inoculation with RKNs. Expression of *JERF3*, *CAT*, and *ACO* genes was detected by quantitative real-time reverse-transcription polymerase chain reaction (qRT-PCR) in roots (A, C, and E, respectively)and leaves (B, D, and F, respectively)of nematode-infested susceptible tomato plants, untreated and BCA-pretreated, 3 and 7 days after inoculation (dpi) with 300 J2*M*. *incognita*. Data are the mean fold changes (n = 6) ±SD in gene transcript levels of tissues from inoculated plants compared with tissues from non-inoculated control plants (the value of 1 indicates no change). An asterisk (*) indicates that the mean fold change is significantly different from 1 as determined by the non-parametric Kolmogorov-Smirnov test (P<0.05).

Conversely, BCA treatment did not affect the systemic inhibition of *JERF3* gene expression induced by nematode infection (Fig 4A and 4B); moreover, *JERF3* gene expression did not change in both untreated and BCA-treated plants 7 days after nematode inoculation. *CAT* gene expression in inoculated roots resulted to be about 7-fold higher than that in uninoculated roots in roots both at 3 and 7 dpi; in this case, BCA treatment resulted in a drastic (3 dpi) or limited (7 dpi) reduction of such an over-expression (Fig 4C). On the contrary, leaves were not involved in the activation of this gene: nematode inoculation to untreated or to BCA-treated plants did not change *CAT* gene expression with respect to healthy control plants (Fig 4D). BCA treatment systemically primed *ACO* gene expression against nematodes (Fig 4E and 4F), as it occurred with the *PR*-genes tested. Also in the absence of BCAs, nematode infection induced an increase of transcript levels of the gene, although much slighter than in their presence.

Chitinase (CHI) and glucanase (GLU) are defense-induced enzymes in plants. Moreover, Reactive Oxygen Species (ROS), such as hydrogen peroxide (H_2_O_2_),are normally produced in response to biotic challenges because anti-microbial. H_2_O_2_ is presumed to orchestrate basal and systemic defense to invading pests. Antioxidant enzymes, such as ascorbate peroxidase (APX), degrade H_2_O_2_ favoring biotic infections. We tested the activity of these three enzymes in roots of untreated and BCA-treated tomato plants at 3 and 7 dpi (Table 3). CHI activity was moderately induced by nematode infection at both 3 and 7 dpi. When plants were pre-treated with BCAs, a more intense induction of this activity was observed. Conversely, GLU activity seems not to be activated by nematode infection; however, if plants were pre-treated with BCAs, a marked increase (+62%) of this activity was caused by nematode infection at 7 dpi. Nematode infection favored the increase of APX activity to maintain low peroxidative reactions which can jeopardize J2 development. BCA pre-treatment was not able to restrain this increment, at least during the earliest stages of infection.

**Table 3:** Effect of RKN inoculation on enzyme activities in roots of tomato plants untreated or pre-treated with BCAsat different days after inoculation (dpi).

Enzyme	Roots untreated	Inoculated Roots untreated	effect %	Roots BCA-treated	Inoculated Roots BCA-treated	effect %
**CHI**^a^						
3 dpi	0.15±0.02	0.19±0.04**	+30	0.22±0.07	0.32±0.15*	+48
7 dpi	0.32±0.11	0.37±0.09*	+15	0.19±0.06	0.28±0.12**	+50
**GLU**^b^						
3 dpi	46.2±8.0	48.6±11.1	ns	51.8±6.4	54.0±11.9	ns
7 dpi	51.8±14.5	65.5±10.2	ns	33.5±10.1	54.4±5.9*	+62
**APX**^c^						
3 dpi	0.24±0.03	0.40±0.04**	+65	0.32±0.03	0.47±0.14*	+46
7 dpi	0.41±0.16	0.54±0.12**	+33	0.24±0.03	0.37±0.04**	+53

significantly different (*P<0.05; **P<0.01) according to a paired *t*-test; ns = not significant;

^a^chitinase expressed as nkat mg^-1^prot;

^b^ glucanase expressed as μmol glucose min^-1^ mg^-1^prot;

^c^ascorbate peroxidase expressed as μmole ascorbate min^-1^ mg^-1^prot

## Discussion

Most of the BCAs used in this study to induce plant immune system were AMF and BCF. Symbiotic fungi colonize plant roots of dicots and monocots, and such interaction has as a consequence the reprogramming of plant transcriptome and proteome [2]. One of the main effect of transcriptomic changes in colonized plants is the elicitation of resistance to a large variety of pathogens and parasites, from fungi to viruses, nematodes included [3, 4]. We analyzed transcript levels of various genes involved in plant defense up to 12 days after a soil-drench treatment of tomato plants with both AMF and BCF. Most of the analyzed genes resulted either up- or down-regulated according to the time span following fungi inoculation, and the response was generally systemic. Among the *PR*-genes tested, *PR-1b* was the earliest and the most highly expressed gene after BCA treatment, as its transcript levels were 2.5/5-fold higher in systemic tissues of BCA-treated with respect to untreated plants. Conversely, *PR-5* gene BCA-induced expression was much lower and was observed later after the treatment than that of *PR-1b*.*PR-1* gene has been referred as the immune marker gene and the target gene for SA [37, 38]; moreover, PR-1 proteins have often been used as markers for SAR, although their biological activity has remained elusive [39]. Both *PR-1* and *PR-5* are commonly used as molecular markers for SA-dependent signaling and have consistently been found to be coordinately regulated by SA [40]. However, it should be noted that some reports have indicated *PR-5* gene as not inducible by SA in tomato [26]. *PR-1* and *PR-5* genes were differently up-regulated in tomato plants treated with SA, as well [34]. At the earliest days after both SA and BCA treatments, a systemic and consistent over-expression of *PR-1* gene occurred, whilst a slight and limited to roots over-expression of *PR-5* gene was observed only at later stages. It was possible to observe, at least for *PR-1* gene, that the initial activation of expression was followed by a drastic inhibition. Expression of *PR-3* gene was generally not affected by BCA treatment; a systemic gene down-loading was detected only at 12 dpt.

AMF secrete suppressors of immunity as a strategy and are sensitive to SA-regulated defenses, which they share with pathogenic fungi [41]. Immunity and symbiosis signaling pathways involve receptor complexes and a variety of processes with notable similarities [42]. Therefore, plants may at first recognize AMF as pathogens and react by activating SAR, as indicated by the high *PR-1* up-regulation shown in Fig 1A. Activation of *CAT* gene expression, as well, may indicate that plants try to produce additional antioxidant enzyme activities to protect themselves from the H_2_O_2_ typically generated in the early response to biotic challenges. However, AMF are able to repress SA-dependent defense in later stages to achieve a compatible interaction [11], as confirmed by data shown herein. An early activation and subsequent repression of gene regulation during fungi colonization characterized the JA/ET-responsive *JERF3* gene, as well. Moreover, *ACO* gene encoding for the key enzyme of ET biosynthesis was also late down-regulated after BCA treatment. It is apparent that a successful colonization may require the inhibition of both SA- and JA/ET-responsive gene expressions. On the contrary, during *Trichoderma*-tomato interaction, repression of defense gene expression was apparent as early as one day after conidia inoculation [8]. In this previous study, it is possible that early defense activation could not be observed, as it is known that the first defense response against *Trichoderma* spp. may occur only one hour after inoculation [2]. In the present study, gene expression during BCA-root interaction generally showed an initial reaction to inoculation by plants followed by a much later defense repression, unraveling a process that shares strict similarities with root mycorrhization.

Mycorrhized plants have been shown to be primed against different pathogens by the so-called MIR [11]. Comparably, our BCA-treated plants were primed against RKNs. In the primed state, the immune system of plants is activated, and plants respond to biotic attacks with faster and stronger defense activation [9]. Immunity expressed against RKNs by BCA-primed plants seems to rely on the up-regulation of various tested genes, such as *PR*- and *ACO* genes. It is interesting to note that the expression of the tested *PR*-genes was markedly and systemically repressed in the untreated plants attacked by nematodes. Priming induced by BCA treatment allows plants to respond to nematodes by a marked activation of the genes encoding for these defense proteins. Such a rapid and strong defense reaction may contribute to drastically lower in BCA-pretreated plants the amount of juveniles able to build a feeding site and develop. It is generally known that SA-responsive genes, such as *PR-1*, are crucial for resistance against biotrophic pathogens and parasites, nematodes included [26, 43]. On the other hand, the endochitinases encoded by *PR-3* have already been associated with resistance conferred by *Glomus versiforme* to grapevine roots against *Meloidogyne incognita* [44]. Comparably, chitinase and glucanase activities were found to be markedly enhanced in roots of BCA-primed plants after nematode inoculation. Systemic up-loading of *ACO* gene suggests that ET level might increase in BCA-primed roots and leaves, upon nematode attack. Actually, ET and ET-signaling have already been reported to play a role in plant defense against endoparasitic sedentary nematodes [45]. SA- and ET-signaling may cooperate for a more efficient and rapid response to nematode infection, confirming that synergistic signaling cross-talks are common in plant resistance [46]. BCA-mediated priming of tomato plants does not seem to involve the activation of the JA-dependent *JERF3* gene. If we consider *JERF3* as a marker gene for the rhizobacteria-mediated ISR, we can reasonably argue that ISR is not activated in BCA-primed tomato plants against RKNs. Conversely, JA-mediated ISR is generally known to activate defense against necrotrophs or herbivorous insects [11].

Although nematodes are able to suppress plant defense, and particularly *PR*-gene expression, for a successful compatible interaction [16, 47, 48], they induce the activation of the plant antioxidant enzyme system to neutralize the toxic ROS generated by basal plant defense. Transcript levels of *CAT* gene were found to be about 7-fold higher in roots of inoculated plants compared with uninoculated controls. For the first time, we found that the MIR, putatively observed in this study, against RKNs involves a marked restraint of this nematode-mediated *CAT* gene up-regulation as early as 3 days after inoculation. A similar early restriction of the nematode-induced APX activity enhancement was detected in roots of primed plants at the same stage after inoculation. Somehow, these effects by BCA-priming seem to be lost at 7 dpi. Both CAT and APX show a H_2_O_2_-degrading activity that favors nematode development; thus, its limitation may augment the chances to contrast parasite invasion by plant roots. SA accumulation in primed plants challenged by nematodes can be predicted by the rapid and extensive over-expression of *PR-1b* gene; exogenously-added SA to tomato plants caused either a very high increase of *PR-1* transcript levels or a high content of endogenous free SA, in both roots and leaves [34, 49]. In turn, SA accumulation leads to H_2_O_2_ accumulation [50]. The maintenance of a highly active anti-oxidant enzyme system despite BCA priming may partly explain the attenuation of the protective BCA-effect over time. Genetic resistance of tomato to RKNs, which does not allow the development of the inoculated juveniles, has been associated to a marked inhibition of root CAT and APX after inoculation [51].

BCA-conferred immunity did not function by a restriction of nematode entry into the root. Evidently, activation of immunity in this type of plant-pest interaction acts by opposing the attempt by the invading J2s to build a feeding siteat the expense of few cortical cells in the root elongation zone. A functional feeding site allows the juvenile to suck nutrients from plant metabolism, to become sedentary and develop into a reproducing female. Only sedentary stages were drastically limited in roots of BCA-treated plants. Elicitation of plant defense machinery in primed plants occurred, in this study, as early as 3 days after inoculation, when only motile forms were found. It is evident that immunity was triggered before feeding site arrangement, when vermiform juveniles are still moving through the elongation zone in search of suitable cortical cells to pierce and feed with their stylet. According to our findings, plant immunity may be as rapid as to be triggered by contact with nematodes. Although supposedly triggered at the first contacts with J2s, immunity likely works in limiting their subsequent feeding site arrangements, thus decreasing the amount of sedentary forms. Once feeding site is somehow successfully arranged, development and reproduction are no longer affected. However, priming may have an effect that tends to reduce over time, as mentioned above. Adult females extracted from primed roots 21 days after inoculation were about 80% less than those from not primed control roots. At 40 days after inoculation, much more individuals were found to have developed up to gravid females, also in primed plants. It can be argued that the many J2s,which had previously entered the roots, although retarded in their development, maysubsequently have the chance to build their feeding site and reproduce. However, the overall protective effect of priming determined about 50% inhibition of infection at the end of experimental time, in terms of less females and egg masses found in roots.

In this study, for the first time, a commercial product, containing a mixture of several beneficial microorganisms, has been used to monitor plant priming, in terms of systemic gene expression activation against nematodes. The bacterial components of this mixture were proved not to be the priming-inducers, because when the BCA mixture was incubated with Amphotericin B, a potent antifungal compound, pre-treatments of plants lost their ability to induce resistance. The involvement of abiotic factors was ruled out by pre-treating plants with sterilized mixture that did not cause any changes in nematode infection (results not shown).

In conclusion, ET- and SA-responsive genes seem to be up-regulated in the activation of plant immune system by beneficial fungi against soil-borne parasites, such as RKNs. Moreover, the enhancement of glucanase and chitinase activities, as well as down-regulation of genes encoding for antioxidant enzymes, were proved to be involved. The immunity conferred is systemic but its effect probably decreases as nematode infection proceeds. On-going experiments with BCA-treated plants infected with miner insects confirm that this type of immunity works also against leaf parasites [52]. Further investigation is needed to promote BCA treatments as an additional strategy in current integrated pest management, because of the complex interactions that such beneficial microorganisms may have with existing soil microbiome of different plant species. However, strategies based on activation of plant innate immunity seem potentially suitable for a low-impact pest management that can be profitable for farmers, diffused in organic agriculture, and compatible with EU agricultural policy.

## Supporting information

S1 Table qRT-PCRs of defense genes from roots and leaves of tomato plants 3, 7, 8, 12 days after treatment (dpt) with BCAs, and of roots and leaves of BCA-pretreated 3 and 7 days after inoculation with 300 juveniles of the root-knot nematode *Meloidogyne incognita*. Each value is expressed as 2^-ΔΔC^t and indicates gene transcript levels of tissues from BCA-treated plants relative to those from untreated control plants, or of tissues from inoculated plants relative to those from not inoculated control plants (the value 1 indicates no change). Each value comes from a single RNA extraction. Three RNA extractions were performed per bioassay; 2 different bioassays were carried out. The mean fold changes are calculated from 6 replicates and shown as means ± standard deviations. An asterisk (*) indicates that the means are significantly different from 1 as determined by the non-parametric Kolmogorov-Smirnov test (*P*<0.05).
(DOCX)
