# Supplementary material for: Bio-control agents activate plant immune response and prime susceptible tomato against root-knot nematodes
Source: PLoS One. 2019 Dec 3;14(12):e0213230. doi: 10.1371/journal.pone.0213230 (PMC6890175; doi:10.1371/journal.pone.0213230)
Supplement: S1 Table — Each value is expressed as 2-ΔΔCt and indicates gene transcript levels of tissues from BCA-treated plants relative to those from untreated control plants, or of tissues from inoculated plants relative to those from not inoculated control plants (the value 1 indicates no change). Each value comes from a single RNA extraction. Three RNA extractions were performed per bioassay; 2 different bioassays were carried out. The mean fold changes are calculated from 6 replicates and shown as means ± standard deviations. An asterisk (*) indicates that the means are significantly different from 1 as determined by the non-parametric Kolmogorov-Smirnov test (P<0.05). (DOCX) [file pone.0213230.s001.docx]

**S 1 Table:** qRT-PCRs of defense genes from roots and leaves of tomato plants 3, 7, 8, 12 days after treatment (dpt) with BCAs, and of roots and leaves of BCA-pretreated 3 and 7 days after inoculation with 300 juveniles of the root-knot nematode *Meloidogyne incognita*. Each value is expressed as 2^-∆∆C^t and indicates gene transcript levels of tissues from BCA-treated plants relative to those from untreated control plants, or of tissues from inoculated plants relative to those from not inoculated control plants (the value 1 indicates no change). Each value comes from a single RNA extraction. Three RNA extractions were performed per bioassay; 2 different bioassays were carried out. The mean fold changes are calculated from 6 replicates and shown as means ± standard deviations. An asterisk (*) indicates that the means are significantly different from 1 as determined by the non-parametric Kolmogorov-Smirnov test (*P*<0.05).

| **gene/dpt** | **Roots BCA-treated/ untreated** | **Leaves BCA-treated/ untreated** | **gene/dpi** | **Roots untreated**  **inoc/non inoc** | **Roots BCA-pretreated**  **inoc/non inoc** | **Leaves untreated**  **inoc/non inoc** | **Leaves BCA-pretreated**  **inoc/non inoc** |
| --- | --- | --- | --- | --- | --- | --- | --- |
| ***PR-1b*** |  |  | ***PR-1b*** |  |  |  |  |
| **3 dpt** |  |  |  |  |  |  |  |
| **BIO1 - RNAex1** | 2.57 | 5.86 |  |  |  |  |  |
| **BIO1 - RNAex2** | 2.87 | 2.73 |  |  |  |  |  |
| **BIO1 - RNAex3** | 2.39 | 6.59 |  |  |  |  |  |
| **BIO2 - RNAex1** | 1.46 | 9.25 |  |  |  |  |  |
| **BIO2 - RNAex2** | 3.58 | 2.50 |  |  |  |  |  |
| **BIO2 - RNAex3** | 3.51 | 5.74 |  |  |  |  |  |
| **Mean ± SD** | 2.73±0.79* | 5.44±2.53* |  |  |  |  |  |
|  |  |  |  |  |  |  |  |
| **7 dpt** |  |  |  |  |  |  |  |
| **BIO1 - RNAex1** | 4.32 | 10.56 |  |  |  |  |  |
| **BIO1 - RNAex2** | 8.11 | 5.98 |  |  |  |  |  |
| **BIO1 - RNAex3** | 4.92 | 9.19 |  |  |  |  |  |
| **BIO2 - RNAex1** | 4.96 | 3.81 |  |  |  |  |  |
| **BIO2 - RNAex2** | 2.60 | 3.41 |  |  |  |  |  |
| **BIO2 - RNAex3** | 2.77 | 2.77 |  |  |  |  |  |
| **Mean ± SD** | 4.61±2.00* | 5.95±3.25* |  |  |  |  |  |
|  |  |  |  |  |  |  |  |
| **8 dpt** |  |  | **3 dpi** |  |  |  |  |
| **BIO1 - RNAex1** | 0.66 | 0.43 |  | 0.10 | 5.98 | 0.27 | 4.06 |
| **BIO1 - RNAex2** | 0.67 | 0.31 |  | 0.12 | 5.74 | 0.31 | 7.01 |
| **BIO1 - RNAex3** | 0.38 | 0.36 |  | 0.14 | 8.06 | 0.31 | 5.74 |
| **BIO2 - RNAex1** | 0.37 | 0.24 |  | 0.67 | 5.74 | 0.59 | 8.57 |
| **BIO2 - RNAex2** | 0.88 | 0.20 |  | 0.61 | 3.07 | 0.42 | 6.41 |
| **BIO2 - RNAex3** | 0.42 | 0.25 |  | 0.50 | 6.68 | 0.33 | 4.00 |
| **Mean ± SD** | 0.57±0.21* | 0.30±0.09* |  | 0.36±0.27* | 5.88±1.63* | 0.37±0.12* | 5.96±1.77* |
|  |  |  |  |  |  |  |  |
| **12 dpt** |  |  | **7 dpi** |  |  |  |  |
| **BIO1 - RNAex1** | 1.13 | 0.95 |  | 0.86 | 8.57 | 0.79 | 3.14 |
| **BIO1 - RNAex2** | 1.14 | 0.68 |  | 0.84 | 5.50 | 0.33 | 5.35 |
| **BIO1 - RNAex3** | 0.51 | 0.46 |  | 0.62 | 8.88 | 0.36 | 4.50 |
| **BIO2 - RNAex1** | 0.35 | 1.68 |  | 0.84 | 7.84 | 0.56 | 2.04 |
| **BIO2 - RNAex2** | 0.35 | 1.59 |  | 0.74 | 5.39 | 0.48 | 2.19 |
| **BIO2 - RNAex3** | 0.46 | 1.21 |  | 0.89 | 5.10 | 0.31 | 2.31 |
| **Mean ± SD** | 0.66±0.38* | 1.09±0.49 |  | 0.80±0.10* | 6.88±1.74* | 0.47±0.18* | 3.26±1.38* |
|  |  |  |  |  |  |  |  |
| **PR-3** |  |  | **PR-3** |  |  |  |  |
| **3 dpt** |  |  |  |  |  |  |  |
| **BIO1 - RNAex1** | 0.87 | 1.37 |  |  |  |  |  |
| **BIO1 - RNAex2** | 1.02 | 0.62 |  |  |  |  |  |
| **BIO1 - RNAex3** | 0.99 | 0.87 |  |  |  |  |  |
| **BIO2 - RNAex1** | 1.14 | 0.38 |  |  |  |  |  |
| **BIO2 - RNAex2** | 0.60 | 0.43 |  |  |  |  |  |
| **BIO2 - RNAex3** | 0.93 | 0.93 |  |  |  |  |  |
| **Mean ± SD** | 0.90±0.18 | 0.77±0.36 |  |  |  |  |  |
|  |  |  |  |  |  |  |  |
| **7 dpt** |  |  |  |  |  |  |  |
| **BIO1 - RNAex1** | 0.73 | 0.74 |  |  |  |  |  |
| **BIO1 - RNAex2** | 0.46 | 0.48 |  |  |  |  |  |
| **BIO1 - RNAex3** | 0.81 | 1.07 |  |  |  |  |  |
| **BIO2 - RNAex1** | 1.11 | 0.15 |  |  |  |  |  |
| **BIO2 - RNAex2** | 1.16 | 0.93 |  |  |  |  |  |
| **BIO2 - RNAex3** | 0.88 | 1.43 |  |  |  |  |  |
| **Mean ± SD** | 0.86±0.26 | 0.97±0.33 |  |  |  |  |  |
|  |  |  |  |  |  |  |  |
| **8 dpt** |  |  | **3 dpi** |  |  |  |  |
| **BIO1 - RNAex1** | 0.30 | 0.42 |  | 2.03 | 3.63 | 2.51 | 3.13 |
| **BIO1 - RNAex2** | 0.16 | 1.16 |  | 1.15 | 2.36 | 2.54 | 3.23 |
| **BIO1 - RNAex3** | 0.94 | 0.93 |  | 1.33 | 2.75 | 1.58 | 1.52 |
| **BIO2 - RNAex1** | 0.96 | 0.41 |  | 1.27 | 5.78 | 1.05 | 1.00 |
| **BIO2 - RNAex2** | 1.71 | 0.84 |  | 1.54 | 4.08 | 0.85 | 1.46 |
| **BIO2 - RNAex3** | 1.20 | 1.01 |  | 1.24 | 4.76 | 1.67 | 1.38 |
| **Mean ± SD** | 0.88±0.57 | 0.80±0.31 |  | 1.42±0.32* | 3.89±1.27* | 1.70±0.71* | 1.72±0.87* |
|  |  |  |  |  |  |  |  |
| **12 dpt** |  |  | **7 dpi** |  |  |  |  |
| **BIO1 - RNAex1** | 0.17 | 0.23 |  | 0.40 | 9.32 | 0.37 | 1.18 |
| **BIO1 - RNAex2** | 0.66 | 0.46 |  | 0.75 | 3.92 | 0.62 | 1.77 |
| **BIO1 - RNAex3** | 0.31 | 0.62 |  | 0.64 | 6.77 | 0.45 | 0.90 |
| **BIO2 - RNAex1** | 0.43 | 0.82 |  | 0.26 | 5.56 | 0.49 | 0.61 |
| **BIO2 - RNAex2** | 0.47 | 0.99 |  | 0.61 | 2.97 | 0.39 | 0.51 |
| **BIO2 - RNAex3** | 0.60 | 0.75 |  | 040 | 4.92 | 0.46 | 1.14 |
| **Mean ± SD** | 0.44±0.18* | 0.64±0.27* |  | 0.51±0.19* | 5.58±2.25* | 0.46±0.09* | 1.02±0.46 |
|  |  |  |  |  |  |  |  |
| **PR-5** |  |  | **PR-5** |  |  |  |  |
| **3 dpt** |  |  |  |  |  |  |  |
| **BIO1 - RNAex1** | 1.71 | 1.04 |  |  |  |  |  |
| **BIO1 - RNAex2** | 1.99 | 1.12 |  |  |  |  |  |
| **BIO1 - RNAex3** | 1.17 | 0.77 |  |  |  |  |  |
| **BIO2 - RNAex1** | 1.10 | 1.39 |  |  |  |  |  |
| **BIO2 - RNAex2** | 0.42 | 1.35 |  |  |  |  |  |
| **BIO2 - RNAex3** | 1.06 | 0.93 |  |  |  |  |  |
| **Mean ± SD** | 1.24±0.55 | 1.10±0.24 |  |  |  |  |  |
|  |  |  |  |  |  |  |  |
| **7 dpt** |  |  |  |  |  |  |  |
| **BIO1 - RNAex1** | 1.13 | 0.56 |  |  |  |  |  |
| **BIO1 - RNAex2** | 0.89 | 0.43 |  |  |  |  |  |
| **BIO1 - RNAex3** | 1.18 | 0.82 |  |  |  |  |  |
| **BIO2 - RNAex1** | 1.01 | 1.21 |  |  |  |  |  |
| **BIO2 - RNAex2** | 1.17 | 1.19 |  |  |  |  |  |
| **BIO2 - RNAex3** | 1.08 | 1.06 |  |  |  |  |  |
| **Mean ± SD** | 1.08±0.11 | 0.88±0.33 |  |  |  |  |  |
|  |  |  |  |  |  |  |  |
| **8 dpt** |  |  | **3 dpi** |  |  |  |  |
| **BIO1 - RNAex1** | 1.30 | 0.63 |  | 0.51 | 7.78 | 0.54 | 1.32 |
| **BIO1 - RNAex2** | 1.37 | 0.82 |  | 0.56 | 6.19 | 0.87 | 2.02 |
| **BIO1 - RNAex3** | 1.25 | 0.81 |  | 0.61 | 6.36 | 0.82 | 2.53 |
| **BIO2 - RNAex1** | 2.17 | 1.44 |  | 0.74 | 3.43 | 1.13 | 2.16 |
| **BIO2 - RNAex2** | 1.39 | 0.77 |  | 0.66 | 3.97 | 0.59 | 5.85 |
| **BIO2 - RNAex3** | 1.09 | 1.09 |  | 0.50 | 5.54 | 0.52 | 2.60 |
| **Mean ± SD** | 1.43±0.38* | 0.93±0.29 |  | 0.60±0.09* | 5.55±1.61* | 0.75±0.24* | 3.03±1.60* |
|  |  |  |  |  |  |  |  |
| **12 dpt** |  |  | **7 dpi** |  |  |  |  |
| **BIO1 - RNAex1** | 1.46 | 0.04 |  | 0.65 | 3.18 | 0.23 | 1.54 |
| **BIO1 - RNAex2** | 1.74 | 0.15 |  | 1.17 | 4.47 | 0.54 | 6.43 |
| **BIO1 - RNAex3** | 1.72 | 0.61 |  | 0.31 | 4.32 | 0.46 | 2.66 |
| **BIO2 - RNAex1** | 1.52 | 0.63 |  | 0.29 | 2.52 | 0.31 | 2.11 |
| **BIO2 - RNAex2** | 0.99 | 0.66 |  | 0.77 | 2.20 | 0.18 | 1.65 |
| **BIO2 - RNAex3** | 1.02 | 0.64 |  | 0.67 | 3.68 | 0.25 | 2.01 |
| **Mean ± SD** | 1.41±0.33* | 0.46±0.28* |  | 0.64±0.33* | 3.39±0.93* | 0.33±0.14* | 2.73±1.85* |
|  |  |  |  |  |  |  |  |
| **JERF3** |  |  | **JERF3** |  |  |  |  |
| **3 dpt** |  |  |  |  |  |  |  |
| **BIO1 - RNAex1** | 1.32 | 1.91 |  |  |  |  |  |
| **BIO1 - RNAex2** | 0.98 | 1.92 |  |  |  |  |  |
| **BIO1 - RNAex3** | 2.10 | 2.75 |  |  |  |  |  |
| **BIO2 - RNAex1** | 0.76 | 1.36 |  |  |  |  |  |
| **BIO2 - RNAex2** | 2.11 | 0.98 |  |  |  |  |  |
| **BIO2 - RNAex3** | 2.35 | 1.27 |  |  |  |  |  |
| **Mean ± SD** | 1.60±0.67* | 1.70±0.63* |  |  |  |  |  |
| **7 dpt** |  |  |  |  |  |  |  |
| **BIO1 - RNAex1** | 0.65 | 1.91 |  |  |  |  |  |
| **BIO1 - RNAex2** | 0.82 | 1.04 |  |  |  |  |  |
| **BIO1 - RNAex3** | 0.95 | 1.04 |  |  |  |  |  |
| **BIO2 - RNAex1** | 0.47 | 1.44 |  |  |  |  |  |
| **BIO2 - RNAex2** | 0.95 | 0.42 |  |  |  |  |  |
| **BIO2 - RNAex3** | 1.38 | 0.73 |  |  |  |  |  |
| **Mean ± SD** | 0.87±0.31 | 1.09±0.52 |  |  |  |  |  |
| **8 dpt** |  |  | **3 dpi** |  |  |  |  |
| **BIO1 - RNAex1** | 0.49 | 0.99 |  | 0.48 | 0.67 | 1.71 | 0.55 |
| **BIO1 - RNAex2** | 0.99 | 0.51 |  | 0.46 | 0.31 | 0.55 | 1.20 |
| **BIO1 - RNAex3** | 0.47 | 0.57 |  | 0.64 | 0.30 | 0.56 | 0.41 |
| **BIO2 - RNAex1** | 0.60 | 0.17 |  | 0.49 | 0.84 | 0.75 | 0.56 |
| **BIO2 - RNAex2** | 1.06 | 0.05 |  | 0.83 | 1.08 | 0.26 | 0.78 |
| **BIO2 - RNAex3** | 0.31 | 0.32 |  | 0.85 | 0.50 | 0.36 | 0.14 |
| **Mean ± SD** | 0.65±0.30* | 0.43±0.33* |  | 0.62±0.18* | 0.62±0.31* | 0.70±0.52* | 0.61±0.36* |
|  |  |  |  |  |  |  |  |
| **12 dpt** |  |  | **7 dpi** |  |  |  |  |
| **BIO1 - RNAex1** | 1.31 | 0.23 |  | 1.58 | 0.72 | 1.16 | 0.96 |
| **BIO1 - RNAex2** | 1.59 | 0.16 |  | 1.15 | 0.68 | 0.70 | 0.95 |
| **BIO1 - RNAex3** | 0.78 | 0.89 |  | 1.72 | 1.64 | 1.40 | 1.24 |
| **BIO2 - RNAex1** | 0.90 | 0.90 |  | 0.92 | 1.59 | 2.08 | 0.80 |
| **BIO2 - RNAex2** | 0.35 | 0.80 |  | 0.45 | 1.62 | 1.13 | 0.97 |
| **BIO2 - RNAex3** | 0.69 | 0.70 |  | 0.56 | 0.77 | 0.60 | 0.70 |
| **Mean ± SD** | 0.94±0.45 | 0.61±0.33* |  | 1.06±0.52 | 1.17±0.49 | 1.18±0.54 | 0.94±0.18 |
|  |  |  |  |  |  |  |  |
| **CAT** |  |  | **CAT** |  |  |  |  |
| **3 dpt** |  |  |  |  |  |  |  |
| **BIO1 - RNAex1** | 1.09 | 2.30 |  |  |  |  |  |
| **BIO1 - RNAex2** | 0.64 | 1.21 |  |  |  |  |  |
| **BIO1 - RNAex3** | 1.40 | 0.88 |  |  |  |  |  |
| **BIO2 - RNAex1** | 0.88 | 2.08 |  |  |  |  |  |
| **BIO2 - RNAex2** | 0.84 | 4.38 |  |  |  |  |  |
| **BIO2 - RNAex3** | 1.41 | 2.93 |  |  |  |  |  |
| **Mean ± SD** | 1.04±0.32 | 2.30±1.26* |  |  |  |  |  |
|  |  |  |  |  |  |  |  |
| **7 dpt** |  |  |  |  |  |  |  |
| **BIO1 - RNAex1** | 1.05 | 1.80 |  |  |  |  |  |
| **BIO1 - RNAex2** | 1.37 | 0.70 |  |  |  |  |  |
| **BIO1 - RNAex3** | 1.73 | 2.00 |  |  |  |  |  |
| **BIO2 - RNAex1** | 3.78 | 1.95 |  |  |  |  |  |
| **BIO2 - RNAex2** | 3.10 | 0.96 |  |  |  |  |  |
| **BIO2 - RNAex3** | 3.78 | 2.43 |  |  |  |  |  |
| **Mean ± SD** | 2.47±1.24* | 1.64±0.67* |  |  |  |  |  |
|  |  |  |  |  |  |  |  |
| **8 dpt** |  |  | **3 dpi** |  |  |  |  |
| **BIO1 - RNAex1** | 2.85 | 1.08 |  | 11.47 | 0.99 | 0.59 | 1.10 |
| **BIO1 - RNAex2** | 1.19 | 1.13 |  | 15.45 | 1.80 | 0.70 | 1.57 |
| **BIO1 - RNAex3** | 1.78 | 1.22 |  | 5.66 | 1.04 | 1.45 | 1.07 |
| **BIO2 - RNAex1** | 1.54 | 0.62 |  | 5.28 | 1.00 | 1.65 | 1.53 |
| **BIO2 - RNAex2** | 1.40 | 1.27 |  | 4.41 | 1.43 | 1.27 | 1.06 |
| **BIO2 - RNAex3** | 2.03 | 1.39 |  | 5.62 | 1.04 | 1.58 | 0.76 |
| **Mean ± SD** | 1.80±0.59* | 1.12±0.27 |  | 7.98±4.45* | 1.22±0.33 | 1.21±0.45 | 1.18±0.31 |
|  |  |  |  |  |  |  |  |
| **12 dpt** |  |  | **7 dpi** |  |  |  |  |
| **BIO1 - RNAex1** | 0.28 | 0.62 |  | 13.00 | 9.92 | 1.36 | 1.32 |
| **BIO1 - RNAex2** | 0.43 | 0.83 |  | 6.23 | 8.06 | 0.72 | 1.24 |
| **BIO1 - RNAex3** | 0.52 | 0.86 |  | 10.78 | 3.97 | 1.67 | 1.77 |
| **BIO2 - RNAex1** | 0.76 | 0.10 |  | 11.47 | 2.53 | 0.85 | 1.27 |
| **BIO2 - RNAex2** | 0.48 | 0.23 |  | 4.63 | 2.53 | 0.77 | 0.77 |
| **BIO2 - RNAex3** | 0.33 | 0.16 |  | 3.84 | 2.57 | 1.33 | 1.08 |
| **Mean ± SD** | 0.47±0.17* | 0.47±0.34* |  | 8.32±3.90* | 4.93±3.25* | 1.12±0.39 | 1.24±0.32 |
|  |  |  |  |  |  |  |  |
| **ACO** |  |  | **ACO** |  |  |  |  |
| **3 dpt** |  |  |  |  |  |  |  |
| **BIO1 - RNAex1** | 1.35 | 2.50 |  |  |  |  |  |
| **BIO1 - RNAex2** | 0.86 | 0.99 |  |  |  |  |  |
| **BIO1 - RNAex3** | 1.00 | 0.95 |  |  |  |  |  |
| **BIO2 - RNAex1** | 0.92 | 1.24 |  |  |  |  |  |
| **BIO2 - RNAex2** | 2.27 | 1.11 |  |  |  |  |  |
| **BIO2 - RNAex3** | 1.66 | 1.45 |  |  |  |  |  |
| **Mean ± SD** | 1.34±0.54 | 1.37±0.58 |  |  |  |  |  |
|  |  |  |  |  |  |  |  |
| **7 dpt** |  |  |  |  |  |  |  |
| **BIO1 - RNAex1** | 0.88 | 0.24 |  |  |  |  |  |
| **BIO1 - RNAex2** | 0.54 | 0.04 |  |  |  |  |  |
| **BIO1 - RNAex3** | 1.34 | 0.68 |  |  |  |  |  |
| **BIO2 - RNAex1** | 1.97 | 0.93 |  |  |  |  |  |
| **BIO2 - RNAex2** | 1.00 | 1.21 |  |  |  |  |  |
| **BIO2 - RNAex3** | 1.62 | 1.35 |  |  |  |  |  |
| **Mean ± SD** | 1.23±0.52 | 0.74±0.53 |  |  |  |  |  |
|  |  |  |  |  |  |  |  |
| **8 dpt** |  |  | **3 dpi** |  |  |  |  |
| **BIO1 - RNAex1** | 0.15 | 0.97 |  | 2.57 | 14.03 | 0.59 | 3.51 |
| **BIO1 - RNAex2** | 0.65 | 1.15 |  | 2.68 | 4.00 | 0.91 | 1.74 |
| **BIO1 - RNAex3** | 0.51 | 0.81 |  | 1.75 | 4.99 | 0.67 | 2.01 |
| **BIO2 - RNAex1** | 0.69 | 2.58 |  | 2.01 | 5.54 | 0.58 | 1.19 |
| **BIO2 - RNAex2** | 0.88 | 1.79 |  | 3.27 | 3.63 | 1.55 | 1.31 |
| **BIO2 - RNAex3** | 0.63 | 0.86 |  | 2.97 | 3.20 | 1.42 | 3.56 |
| **Mean ± SD** | 0.59±0.24* | 1.36±0.70 |  | 2.54±0.57* | 5.90±4.07* | 0.95±0.43 | 2.22±1.06* |
|  |  |  |  |  |  |  |  |
| **12 dpt** |  |  | **7 dpi** |  |  |  |  |
| **BIO1 - RNAex1** | 0.75 | 1.39 |  | 1.00 | 18.38 | 2.51 | 2.22 |
| **BIO1 - RNAex2** | 0.47 | 1.42 |  | 0.56 | 13.27 | 2.71 | 3.25 |
| **BIO1 - RNAex3** | 0.80 | 0.42 |  | 0.71 | 9.85 | 1.46 | 6.92 |
| **BIO2 - RNAex1** | 1.18 | 0.21 |  | 2.31 | 2.79 | 1.57 | 7.46 |
| **BIO2 - RNAex2** | 0.82 | 0.25 |  | 1.01 | 4.99 | 2.16 | 5.86 |
| **BIO2 - RNAex3** | 0.57 | 0.31 |  | 1.10 | 8.82 | 1.60 | 5.17 |
| **Mean ± SD** | 0.76±0.25* | 0.67±0.58* |  | 1.12±0.62 | 9.68±5.64* | 2.00±0.53* | 5.15±2.06* |
|  |  |  |  |  |  |  |  |
